# Audio Motor Training at the Foot Level Improves Space Representation

**DOI:** 10.3389/fnint.2017.00036

**Published:** 2017-12-18

**Authors:** Elena Aggius-Vella, Claudio Campus, Sara Finocchietti, Monica Gori

**Affiliations:** Unit for Visually Impaired People (U-VIP), Center for Human Technologies, Fondazione Istituto Italiano di Tecnologia, Genoa, Italy

**Keywords:** rehabilitation, spatial cognition, blind, multisensory integration, hearing

## Abstract

Spatial representation is developed thanks to the integration of visual signals with the other senses. It has been shown that the lack of vision compromises the development of some spatial representations. In this study we tested the effect of a new rehabilitation device called ABBI (Audio Bracelet for Blind Interaction) to improve space representation. ABBI produces an audio feedback linked to body movement. Previous studies from our group showed that this device improves the spatial representation of space in early blind adults around the upper part of the body. Here we evaluate whether the audio motor feedback produced by ABBI can also improve audio spatial representation of sighted individuals in the space around the legs. Forty five blindfolded sighted subjects participated in the study, subdivided into three experimental groups. An audio space localization (front-back discrimination) task was performed twice by all groups of subjects before and after different kind of training conditions. A group (experimental) performed an audio-motor training with the ABBI device placed on their foot. Another group (control) performed a free motor activity without audio feedback associated with body movement. The other group (control) passively listened to the ABBI sound moved at foot level by the experimenter without producing any body movement. Results showed that only the experimental group, which performed the training with the audio-motor feedback, showed an improvement in accuracy for sound discrimination. No improvement was observed for the two control groups. These findings suggest that the audio-motor training with ABBI improves audio space perception also in the space around the legs in sighted individuals. This result provides important inputs for the rehabilitation of the space representations in the lower part of the body.

## Introduction

From childhood, the brain is constantly stimulated by different sensory information coming from the external world. Vision has a predominant role in the development of spatial cognition (Pasqualotto and Proulx, 2012; Gori, 2015). In agreement with this idea, it has been found that blind people are impaired in some aspects of sound localization, such as the localization of end point of a dynamic sound (Finocchietti et al., 2015b), the audio space bisection (Gori et al., 2014), the evaluation of the absolute distance (Kolarik et al., 2013b), the auditory spatial representations of the extrapersonal space in both: reverberant and anechoic environments, for speech, music and noise signals (Kolarik et al., 2017) and the vertical localization of a sound source (Zwiers et al., 2001). On the other hand, it has been shown that the absence of vision, as in blind individuals, improves other auditory skills, such as horizontal sound localization (Lessard et al., 1998; King and Parsons, 1999; Röder et al., 1999; Gougoux et al., 2004; Doucet et al., 2005; Lewald, 2007) and relative distance discrimination (Voss et al., 2004; Kolarik et al., 2013a). The reason why some auditory spatial skills are enhanced and other impaired in blind individuals is still an open question. Similarly, the effect of sensory loss on cortical activity is still matter of debate. Some studies reported that when the most appropriate sense for a specific ability is lacking, such as vision in spatial cognition, the silent pre-existing connection is revealed and leads to new strong connections (Amedi and Meijer, 2005; Dahmen and King, 2007). This thesis is supported by several imaging studies (Paus, 1996; Gougoux et al., 2005; Voss et al., 2006; Martuzzi et al., 2007; Eckert et al., 2008; Frasnelli et al., 2011). However, other imaging studies provided an evidence for reduced connectivity between visual and auditory systems, as well as between visual and somatosensory systems (Liu et al., 2007; Yu et al., 2012; Burton et al., 2014), supporting instead the idea that these heightened abilities reflect re-programming of visual cortex for “metamodal” purpose (Burton et al., 2014).

These findings support the idea indicating that the lack of visual experience interferes with the development of some spatial representations. Multiple rehabilitation procedures and devices have been developed to date to improve inclusion of blind individuals by exploiting audio and tactile channels. We have recently developed a new device called ABBI (The Audio Bracelet for Blind Interaction; Finocchietti et al., 2015a; Gori et al., 2016; Ben Porquis et al., 2017). ABBI is an audio bracelet that provides audio feedback to body movement. Recent results from our group suggest that the use of ABBI improves mobility and spatial cognition in visually impaired children and adults (Cappagli et al., 2017; Finocchietti et al., 2017). This result is in agreement with previous works which have shown that sensory-motor learning is not sensory-modality-specific, but that a novel sensory-motor information can be transferred between sensory modalities (Levy-Tzedek et al., 2012). We can speculate that the use of ABBI could allow the creation of a strict link between auditory and motor signals. The new sensory (audio) feedback to body movement might create a bridge between body and external representations in blind individuals by helping the creation of more complex spatial representations of the environment. This idea is in agreement with recent studies showing that in blind individuals the body can be used as a spatial reference to improve audio spatial representations (Vercillo et al., 2017).

While previous works from our group mainly focused on the recalibration of spatial representations around the upper body portion of space in blind individuals (Finocchietti et al., 2017), no studies have investigated whether the use of this device can be also useful to improve spatial representations around the lower body part in sighted individuals. Improvement of space representation at the lower body part would be important for the rehabilitation of locomotion and legs mobility functions in individuals with motor disabilities. With the aim of improving space representation around the lower body portion in sighted individuals, here we studied their audio space representation before and after a training with ABBI positioned on the subject’s foot.

In order to investigate an improvement of audio spatial precision, we used an audio task for humans that is the front-back sound discrimination. Front-back spatial perceptual ambiguity is known as the cone of confusion (Wallach, 1938), an imaginary cone extending outward from each ear, representing sound source locations producing the same interaural differences. It has been shown that head movements help in discriminating front from back sounds, as it affects inter temporal delay (ITD) and inter level difference (ILD; Wightman and Kistler, 1999). An audio front-back discrimination task around the legs was performed in all subjects before and after the training by asking the subjects to judge if a sound was delivered in the frontal or back space. Forty five sighted subjects, split into three groups, performed two sessions of an audio localization task. The experimental group performed 2 min of audio motor training with ABBI between the two audio tests, while no audio motor training was performed by the control groups, where subjects completed just 2 min of free leg movement without sound, or 20 min of passive sound’s hearing. We expected that only the experimental group improve in localizing sounds after the training with the ABBI device, suggesting that an audio-motor training delivered at foot level improves the spatial representation around the legs. Our results support our hypothesis by showing an improvement only in the experimental group. These results suggest that, as hypothesized, the integration of self-generated sounds with proprioceptive-motor information could be used by our brain to improve spatial representation around the legs. These findings open new possibilities for the use of sensory motor trainings in people with spatial and mobility impairments at the leg level.

## Materials and Methods

### Subjects

Forty five participants were enrolled in the study. Subjects were randomly split into three age (*F*_(2,42)_ = 0.13, *P* = 0.87) and height (*F*_(2,42)_ = 1.35, *P* = 0.37) matched groups: experimental group, which did the audio motor training (*N* = 15; 11 females, age: 26 ± 5, years old, height: 165 ± 9) cm; motor control group, which did only motor training (*N* = 15; 5 females, age: 27 ± 6 years old, height: 170 ± 2) cm and audio control group, which did only audio training (*N* = 15; 7 females, age 26 ± 3 years old, height:170 ± 1) cm. All the participants had a similar educational background, no cognitive impairments, were right handed, and they reported to haven’t any hearing impairment (we administer an online hearing test to be sure all participants had the same hearing perception). The participants provided written informed consent in accordance with the Declaration of Helsinki. The study was approved by the ethics committee of the local health service (Comitato Etico, ASL3 Genovese, Italy).

### Set-Up and Sound Localization Task

**Figure 1:**
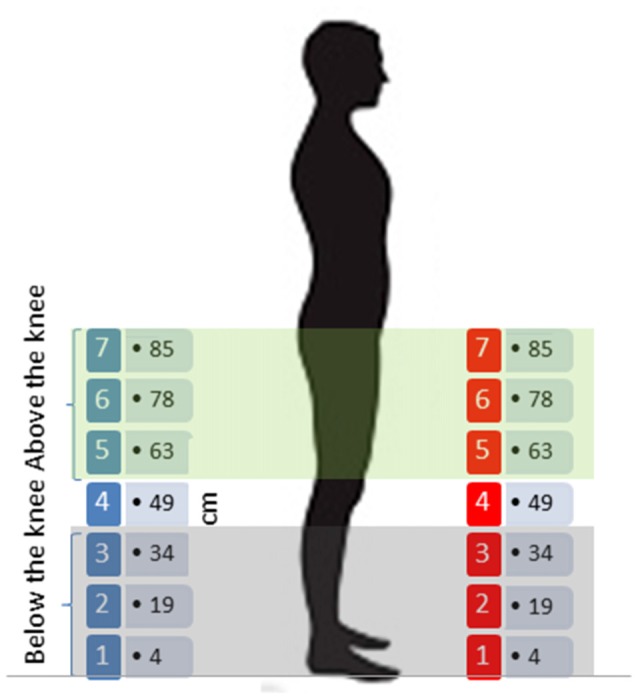
Sound localization task: 14 speakers split into two arrays of seven speakers each, vertically oriented; speakers of each array were positioned at 4 cm, 19 cm, 34 cm, 49 cm, 63 cm, 78 cm and 85 cm from the floor, creating seven equivalent sound elevations in the frontal and rear space. The two arrays were positioned facing each other, one in the frontal space and the other in the rear space.

The experiment was performed in the center of the same dark reverberant room. All participants were positioned in the middle of the room, far from each wall, so that reverberant noise was the same across subjects. As shown in Figure 1, the apparatus consisted of 14 speakers split into two arrays of seven speakers each, vertically oriented; the lowest speaker of each array was positioned at 4 cm from the floor, while the others were situated at: 19 cm, 34 cm, 49 cm, 63 cm, 78 cm, the highest being at 85 cm. There were therefore seven equivalent sound elevations in the frontal and rear space. The two arrays were positioned facing each other; one array of speakers was placed in the frontal space (at 40° in relation to the face) and the other one in the rear space (at 160° in relation to the face); both arrays were situated at a distance of 50 cm from the subject’s position. During each trial, pink noise lasting 1 s was randomly delivered from one of the 14 speakers. Each speaker delivered the sound in six trials, for a total of 84 trials for each session (42 trials in the frontal space and 42 in the rear space). As our goal was to clarify the representation of auditory space around the legs, we split the seven equivalent speakers into two areas: above the knee space and below the knee space, as shown in Figure 1. Above the knee space referred to speakers (numbers 5, 6 and 7-up to 34 cm), while below the knee space (speaker number 4) was represented by (speakers 1, 2 and 3-under 34 cm). We decided to use the knee because it divides the leg into two separate segments, allowing free movement. The knee is also involved in walking and leg actions, and so could influence spatial representation of the two leg segments.

Participants were blindfolded and led into the experimental room, where they remained standing for the entire session (they were allowed to rest before the training). They were asked to keep their head straight and not to direct it toward the sound. They had to verbally report if sounds were delivered in the frontal or in the back area, without considering their spatial elevations. Subject position and posture were continuously monitored and corrected when necessary by the experimenter. Sounds were administered by a custom-made code in Matlab (R2013a, The Math Works, USA); the experimenter recorded on text the oral answer given by the subject (“Front” or “Back”) for the consequent analysis. The entire experiment was performed at the participant’s own pace and each trial started after the subject’s answer, without any time constraints.

### Protocol

The auditory localization task, as previously described, was performed in two sessions (about 20 min each), spaced out by 2 min of training (Figure 1).

The experimental group underwent audio-motor training with the sound source (digital metronome with single pulse 500 Hz, intermittent sound at 180 bpm), delivered by ABBI, placed on the left ankle; they were asked to move their left leg and consequently the sound, from the frontal position to the rear and vice versa, to freely explore space around the body. It was required a continuous and constant movement. The short timing for the audio-motor training was chosen because a previous study (Finocchietti et al., 2017) showed that the spatial recalibration is fast, thanks to the association of the auditory feedback with a voluntary movement. Two control conditions were performed. One control group (motor control group) performed only the same free leg movement, by repeatedly moving the leg from the front position to the back position (as in the group trained with ABBI) but without audio feedback associated to the movement. The second control group (audio control group) listened to the ABBI sound moved by the experimenter with known position. In this case, the experimenter provided before the sound in front and afterwards in the back (random order) by communicating to the subject the spatial position of the sound: the sound was presented for 1 min in the front and for 1 min in the back. The subjects of this group received the same amount of sound feedback as the experimental group. After the training, all groups performed the second session of the sound localization task.

### Data Analysis and Statistics

**Figure 2:**
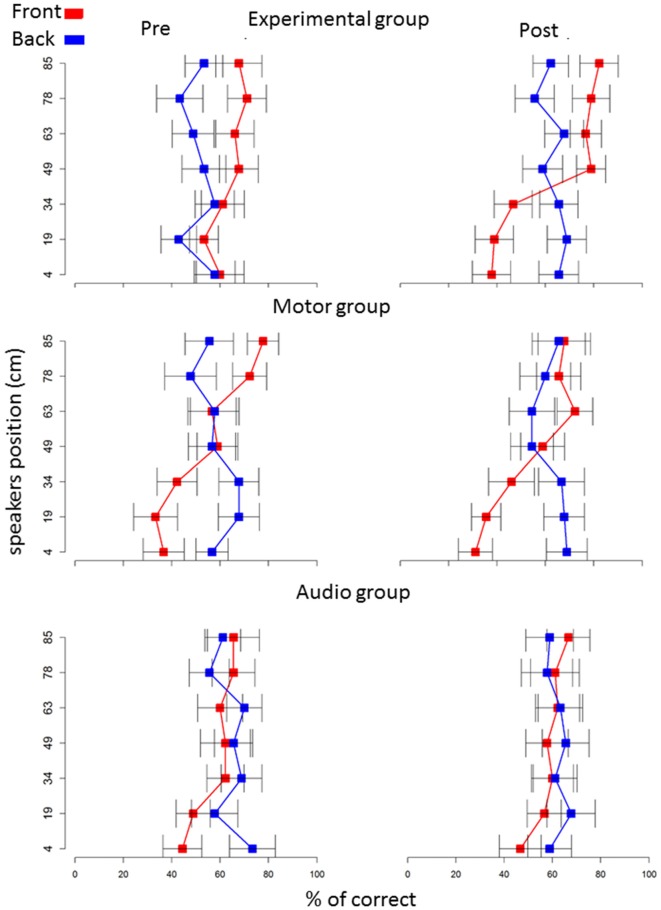
Percentage of correct answers on each speaker: red squares represents frontal speakers, blue squares represents back speakers. Plots represent percentage of correct answers (*x* axis) in the pre (left column) and post (right column) session.

Localization data were post-processed and analyzed by a custom made program in R (R Development Core Team, New Zealand). We removed speaker number 4 (49 cm) from the analysis, as it was at the edge between space below and above the knee level. The six sound sources remained were grouped into two spatial levels: below the knee (speaker numbers 1, 2 and 3) and above the knee (speaker numbers 5, 6 and 7), *t* test confirmed no differences inside these two spatial portions (Figure 2). In order to evaluate the relation between sound localization and the role of senses in representing spaces, we analyzed the pool of single trials using generalized linear mixed models (GLMMs). In this way, we could estimate the variability of fixed and random effects (Moscatelli et al., 2012). We applied GLMM with a logit link function and a binomial distribution. Our model was random-slope (or maximal) following Barr guidelines (Barr et al., 2013; Barr, 2013) and was set for all subjects, taking into account the individual variability in the responses. We set the model to the choices from the localization task using the lme4 package (Bates et al., 2015) in the R statistical language. The model took into account the correct response; to do this, we regressed, in each trial, the answers of each subject considered the correct answer (1 = correct, 0 = incorrect), as a function of sound level (above the knee vs. below the knee), longitudinal position (front vs. back space) and session (pre vs. post) as factors within subjects, while group (experimental vs. motor control and vs. audio control) as factor between subjects. These factors are included in our model as fixed effects. We calculated Analysis of Deviance Tables (using Type II Wald chi-square tests) for the models using the analysis of variance (ANOVA) function of the car package (Fox and Weisberg, 2011). For significant effects, we performed *post hoc* comparisons using lsmeans package (Lenth, 2016), which computes and contrasts least-squares means (predicted marginal means). We adopted Holm P adjustment. Contrasts with *P* < 0.05 were considered as significant (P corrected are reported). Data are presented as mean ± standard error.

## Results

**Figure 3:**
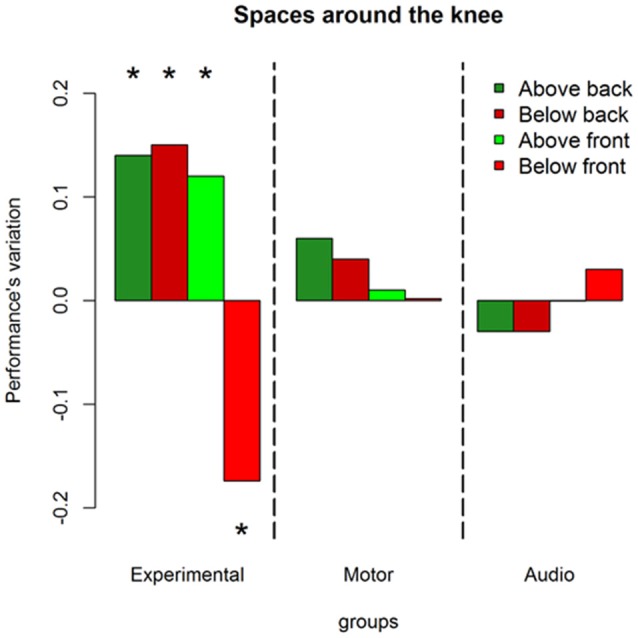
Performance’s variation in each space after the training. Green bars represent space above the knee, red bars denote space above the knee. Dark colors are used for the back space, while light colors are used for the frontal area. As can be seen only the experimental group shows performance’s variations (post—pre) after the training, leading to an improvement in the back space and to a worsened performance in the frontal space under the knee. *Indicates *P* < 0.05.

Results on the analysis of deviance showed a multiple interaction between longitudinal space (front vs. back), sound level (above the knee vs. below the knee), session (pre vs. post) and groups (experimental, motor and audio control) $X_{\left(2\right)}^{2}$ = 11.86, *P* = 0.002. Figure 3 shows this interaction in terms of performance’s variations, i.e., the difference (post session-pre session) of the probability to respond correct calculated by the lsmens function for the *post hoc* contrasts based on the GLMM model (Prob in Table 1). Table 1 reports standard errors and confidence intervals estimated for the pre and post session. Green bars represent sounds delivered above the knee level, red bars represent sounds delivered below the knee level; light colors denote sounds delivered in the frontal space, while dark colors denote sounds delivered in the back space. Positive values of the bars represent improvement in performance in the post session compared to the pre session, and negative values represent decrement in performance. As can be seen, only the experimental group showed performance’s variations after the training. Specifically, considering the back area, an improvement is present in space above the knee (dark green bar; (OR) = 1.7 ± 0.36, z.ratio = 2.9, *P* = 0.01) and below the knee (dark red bar; (OR) = 1.91 ± 0.4, z.ratio = 2.8, *P* = 0.01). Instead, in the frontal space, an improvement is visible above the knee (light green bar; (OR) = 2.04 ± 0.5, z.ratio = 2.5, *P* = 0.02), while a performance worsened below the knee (light red; (OR) = 0.48 ± 0.09, z.ratio = 3.8, *P* = 0.0006). Therefore, performance’s variations in the frontal but not in the back space were strongly dependent on the elevation at which sounds were delivered.

**Table 1:** Interaction between longitudinal position, sound level (above the knee, below the knee), groups (experimental, motor control and audio control) and session (pre, post): table reports probability to be correct, in the pre and post session, in each space for each group.

EXPERIMENTAL						
Session	Sound level	Position	Prob	SE	asymp.LCL	asymp.UCL
Post	Above knee	Back	0.63	0.05	0.06	0.74
Pre	Above knee	Back	0.49	0.06	0.07	0.62
Post	Below knee	Back	0.68	0.05	0.05	0.78
Pre	Below knee	Back	0.53	0.04	0.05	0.62
Post	Above knee	Front	0.83	0.04	0.04	0.90
Pre	Above knee	Front	0.71	0.05	0.05	0.81
Post	Below knee	Front	40.0	0.04	0.04	0.49
Pre	Below knee	Front	0.58	0.04	0.04	0.67
**MOTOR CONTROL**
Post	Above knee	Back	0.62	0.06	0.50	0.72
Pre	Above knee	Back	0.55	0.07	0.42	0.67
Post	Below knee	Back	0.70	0.05	0.59	0.79
Pre	Below knee	Back	0.65	0.04	0.56	0.73
Post	Above knee	Front	0.72	0.06	0.59	0.82
Pre	Above knee	Front	0.72	0.05	0.60	0.81
Post	Below knee	Front	0.37	0.04	0.29	0.45
Pre	Below knee	Front	0.37	0.04	0.29	0.45
**AUDIO CONTROL**
Post	Above knee	Back	0.62	0.06	0.50	0.72
Pre	Above knee	Back	0.65	0.06	0.52	0.76
Post	Below knee	Back	0.64	0.05	0.53	0.74
Pre	Below knee	Back	0.68	0.04	0.59	0.76
Post	Above knee	Front	0.66	0.07	0.52	0.78
Pre	Above knee	Front	0.66	0.06	0.54	0.77
Post	Below knee	Front	0.55	0.04	0.46	0.63
Pre	Below knee	Front	0.52	0.04	0.43	0.60

*Asymp.LCL and asymp.UCL report the control limits (lower and upper respectively). They represent the range of expected variation*.

**Figure 4:**
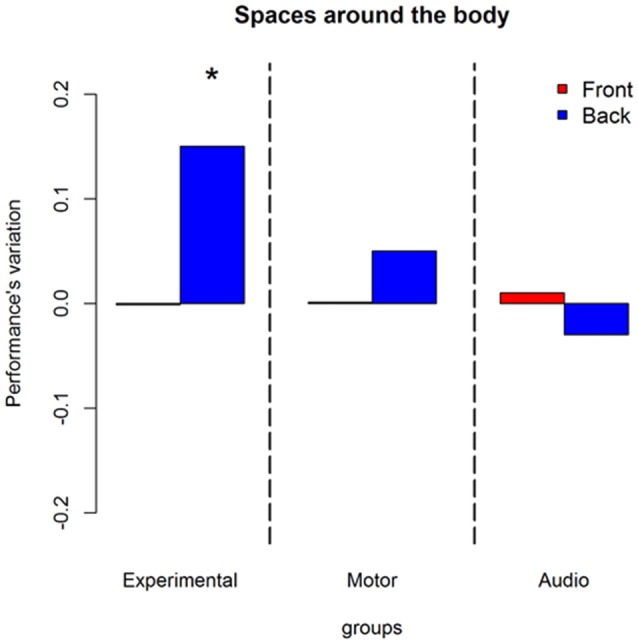
Influences of training on longitudinal position: represent performance’s variation (post—pre) in discriminating front-back location without considering body elevation. Red bars denote frontal sounds, blue bars represent back sounds. As can be seen only the experimental group improved. The improvement is present only in the back space. *Indicates *P* < 0.05.

A second interaction was found between longitudinal position, session and groups $X_{\left(2\right)}^{2}$ = 10.90, *P* = 0.004. Figure 4 explains this interaction in terms of performance’s variations. Blue bars represent back space, while red bars frontal space. As can be seen, an improvement is present only in the experimental group and only in the back space ((OR) = 1.85 ± 0.2, z.ratio = 3.94, *P* = 0.0005).

**Figure 5:**
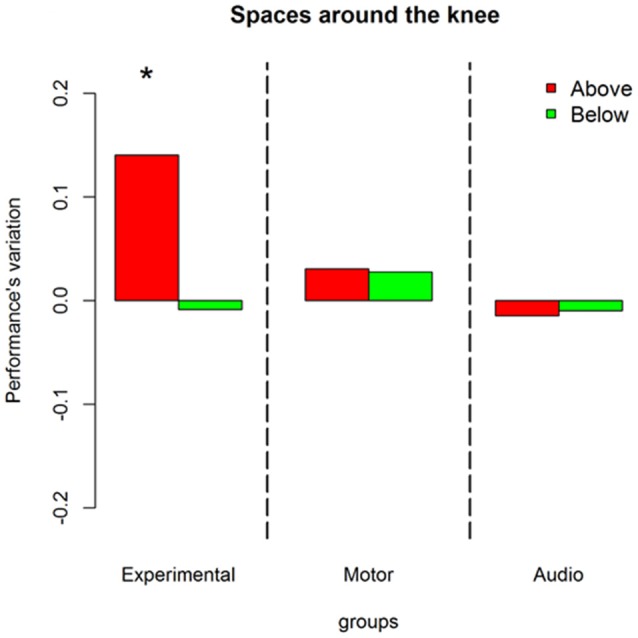
Influences of training on sound elevation: represent performance’s variation (post—pre) in discriminating front-back location at different body elevation. Red bars denote sounds above the knee, green bars represent sounds below the knee. As can be seen only the experimental group improved. The improvement is confined to sounds above the knee. *Indicates *P* < 0.05.

Moreover, a third interaction was found between sound level, session and groups $X_{\left(2\right)}^{2}$ = 8.02, *P* = 0.01. Figure 5 describes this interaction in terms of performance’s variations. Red bars represent sounds delivered above the knee and green bars sounds delivered below the knee. An improvement is present only in the experimental group and only for stimuli presented above the knee ((OR) = 1.91 ± 0.32, z.ratio = 3.77, *P* = 0.0009).

## Discussion

In this study, we tested whether a sensory-motor training, with a new rehabilitative device called ABBI, could be useful to improve the space representation around the lower body part in sighted individuals. Previous studies in blind individuals have shown that a training of few minutes with the ABBI device, a bracelet that produces audio feedback of body movements, improves the audio spatial representation of space around the upper part of the body (Finocchietti et al., 2017). Here we show that a training with ABBI can also improve audio spatial representation in sighted individuals in the lower body space (around the legs). Subjects were asked to perform a front-back sound discrimination task before and after different training conditions. Front-back sound discrimination is a difficult task due to the presence of the cone of confusion. In agreement with previous results (Wenzel et al., 1993), all subjects were around chance level for the front-back sound localization before the trainings. After the training only the experimental group that received the audio motor feedback, by using ABBI, improved spatial performance. No improvement was observed in the other two groups of subjects which performed the training by moving the leg without sound or by listening to the sound moved by the experimenter. These results suggest that only the combination of audio feedback associated with body movement is useful to improve audio spatial representation around the legs in sighted individuals. Future studies will be necessary in order to investigate whether the same is true also for the upper body part since at the moment, in this body region, ABBI has been tested just considering the availability of both audio and motor signals available (Finocchietti et al., 2015a, 2017).

An interesting result is that the effect of training with ABBI varies according to the body parts (above the knee vs. below the knee) and spatial areas (front vs. back) considered. As regard the body parts considered, we observed an improvement above but not below the knee for frontal sounds. The different impact of the audio-motor training on high and low spatial body representation can be explained by considering how often the auditory feedback is linked to those body parts. Indeed when walking, only body space around the foot is mapped by hearing, thanks to the audio feedback produced by the foot reaching the floor: this might be automatically linked to the tactile and proprioceptive information used to encode the leg spatial position. The training with the ABBI device might be less beneficial in the lower portion of the body because at the foot level a natural audio-motor association is already present and it is mediated by locomotion. Another possible speculation is that different multisensory processing act above and below the knee. During locomotion we usually look in front of our feet so visual experience occurs independently respect to the audio-proprioceptive integration related to feet. Since experience can modulate audio-visual integration (Meredith and Stein, 1996), it is plausible that the audio information associated with walking is integrated with proprioceptive feedback on the same spatial area but with visual information congruent in time and not in space. Thus a possible speculation is that this sensory misalignment could lead to distorted or less automatic sensory integration. The training with ABBI might reinforce this misaligned association. The same distortion is not present above the knee because in this body zone the audio feedback of movements isn’t present and multisensory integration is similar to the upper body part where the sensory-motor training with ABBI is useful for spatial recalibration (Finocchietti et al., 2017). As regard the spatial areas considered, several studies indicate that space is processed differently depending both: the body part considered (Serino et al., 2015; di Pellegrino and Làdavas, 2015) and on the distance from the body (Làdavas and Farnè, 2004; Aimola et al., 2012; Tomasino et al., 2012; Caçola et al., 2013; Mahayana et al., 2014). Other studies have found a difference between frontal and rear space (Kóbor et al., 2006; Occelli et al., 2011; Van der Stoep et al., 2015), showing a higher saliency of sounds in the back (Farnè and Làdavas, 2002). Nonetheless only few studies investigated spatial perception around the foot (Schicke et al., 2009; Smid and den Otter, 2013) even if auditory feedback is most commonly perceived at the foot level, for example during locomotion. For different regions around the lower body part (e.g., the foot and upper leg portion), we found different audio performance and recalibration after the audio-motor training supporting the idea that space around the legs is split into sub regions, probably based on the different sensory and motor feedback commonly available in these zones. Indeed, we found an improvement in the back space at both elevation: above and below the knee, suggesting that in space where vision is not available, an audio motor training is useful to recalibrate auditory space. This result is in agreement with other evidences showing the beneficial effect of ABBI in improving auditory space in blind people (Finocchietti et al., 2015a, 2017). For what regards the frontal space, we found a different performance above and below the knee, supporting our hypothesis that these two regions rely on different mechanisms of audio visual integration.

Why the training with the ABBI device is useful to improve audio spatial representations? ABBI favors the association of sensorimotor association and thus it facilitates multisensory integration. Previous studies have shown that audio-motor associations are easily encoded by our brain and transferred across senses (Levy-Tzedek et al., 2012). The flow of information between auditory and motor cortex seems to be bidirectional, and arbitrary sounds (without a previous motor or verbal meaning) can be rapidly mapped onto the motor system (Ticini et al., 2012). Importantly, we observed that the audio-motor training with ABBI improved audio spatial performances. This result seems to be supported by previous studies showing that self-produced stimuli are processed differently than not self-produced stimuli. For example at a behavioral level, self-produced tactile stimulation is perceived as less intense compared with identical tactile stimulation produced by an external source (Blakemore et al., 1999). Similarly, in the auditory modality, when subjects compare the volume of two identical sounds, one self-generated (by actively pressing a button) and the other perceived passively, the self-generated sound is reported as being less loud (Weiss et al., 2011). At the cortical level, self-generated sound activate the sensory cortex differently respect to external sounds (Sato, 2008; Baess et al., 2009, 2011). Crucially, the intention and voluntary aspect of the movement are needed to modulate activity in auditory cortex (Haggard and Whitford, 2004); in other words, the modulation of neural responses to sensory consequences of self-generated actions are influenced by volition and the sense of agency (Haggard, 2008). Since some studies suggest that in case of sensory loss, the silent pre-existing connections are revealed (Amedi et al., 2005; Dahmen and King, 2007), a possible speculation to interpret the improvement observed with ABBI is that the use of self-generated sounds may enhance audio motor integration by unmasking and training pre-existing silent connections, leading to greater effectiveness of this feedback in perceiving space.

The natural feedback provided by the ABBI device has the advantage of being immediate as the subject doesn’t need to use codes for interpreting the sensory feedback he receives, contrarily to what is required by most of the sensory substitution devices developed to date (Cuturi et al., 2016; Gori et al., 2016). Previous results showed that intentional movement has an influence on spatial cognition (Paillard, 1991; Berti and Frassinetti, 1996; Scandola et al., 2016). Our results further confirmed this hypothesis by demonstrating that by adding an auditory feedback to self-generated movements spatial skills improve in spaces unexplored by leg movements, such as above the knee space. Importantly, in this study the improvement is observed also in sighted individuals suggesting that not only blind individuals can benefit from this form of audio-motor feedback (as previously shown in Finocchietti et al., 2017). This effect could be explained by the fact that the sound is integrated with the part of the body that is producing the body movement (i.e., the tight), hence the portion of space calibrated is around the effector driving the motor execution.

To conclude, we showed that an audio motor training below the knee modifies the representation of space around the leg, probably by impacting on different multisensory integration processes. This could explain the improvement and decrement in performance in different zones around the legs. Future experiments will be performed to explore the brain plasticity of the recalibration mediated by the use of ABBI and its application in people with motor disability.

## Author Contributions

EA-V, MG, CC and SF conceived the studies and designed the experiments, wrote the manuscript. EA-V carried out experiments. EA-V and CC analyzed data. All authors reviewed the manuscript.

## Conflict of Interest Statement

The authors declare that the research was conducted in the absence of any commercial or financial relationships that could be construed as a potential conflict of interest.
